# Chloride Diffusion Property of Hybrid Basalt–Polypropylene Fibre-Reinforced Concrete in a Chloride–Sulphate Composite Environment under Drying–Wetting Cycles

**DOI:** 10.3390/ma14051138

**Published:** 2021-02-28

**Authors:** Yang Luo, Ditao Niu, Li Su

**Affiliations:** 1 Department of Civil Engineering, Xi’an University of Architecture and Technology, Xi’an 710055, China; luoyang@xauat.edu.cn (Y.L.); niuditao@xauat.edu.cn (D.N.); 2 State Key Laboratory of Green Building in Western China, Xi’an University of Architecture and Technology, Xi’an 710055, China

**Keywords:** hybrid basalt–polypropylene fibre, chloride–sulphate combined attack, drying–wetting cycles, chloride diffusion property

## Abstract

The effect of fibre reinforcement on the chloride diffusion property of concrete is controversial, and the coupling effect of sulphate erosion and drying–wetting cycles in marine environments has been neglected in previous studies. In this study, the chloride diffusion property of hybrid basalt–polypropylene fibre-reinforced concrete subjected to a combined chloride–sulphate solution under drying–wetting cycles was investigated. The effects of basalt fibre (BF), polypropylene fibre (PF), and hybrid BP–PF on the chloride diffusion property were analysed. The results indicate that the presence of sulphate inhibits the diffusion of chloride at the early stage of erosion. However, at the late stage of erosion, sulphate does not only accelerate the diffusion of chloride by causing cracking of the concrete matrix but also leads to a decrease in the alkalinity of the pore solution, which further increases the risk of corrosion of the reinforcing steel. An appropriate amount of fibre can improve the chloride attack resistance of concrete at the early stage. With the increase in erosion time, the fibre effectively prevents the formation and development of sulphate erosion microcracks, thus reducing the adverse effects of sulphate on the resistance of concrete to chloride attack. The effects of sulphate and fibre on the chloride diffusion property were also elucidated in terms of changes in corrosion products, theoretical porosity, and the fibre-matrix interface transition zone.

## 1. Introduction

The marine economy has become an important part of the global gross national product, and the construction of marine projects such as offshore oil and gas fields, ports, and cross-sea bridges is underway. In marine engineering, reinforced concrete is still the most commonly applied engineering material because of its excellent performance and low cost. However, the marine environment contains a large amount of chloride, which corrodes the reinforcing steel in concrete. This seriously affects the durability of marine engineering structures, resulting in considerable economic losses [1]. Therefore, the selection of construction materials for the severe marine environment should be performed carefully. To reduce the defects of ordinary concrete, an appropriate amount of fibre can be blended into it. The fibre can increase the toughness, tensile strength, and impact resistance of concrete, thus improving the crack resistance of concrete to some extent [2,3].

Fibre-reinforced concrete has been widely used in the past few decades, especially steel fibre, which is widely used in decentralized reinforcement of industrial floors and pavements [4]. Additionally, with the development of fibre materials, there are more and more fibre types to choose from, such as basalt fibre, carbon fibre, polypropylene fibre, polyethylene fibre, polyvinyl alcohol fibre, et cetera. This provides more options for different application environments and conditions. In addition, some studies point out that the bonding between the fibre and the matrix can be effectively improved by changing the shape of the fibre, thus improving the efficiency of the fibre. Marcalikova et al. [5] compared the effect of different amounts of hooked and straight fibres on the mechanical properties of concrete and the results showed that the increase in fracture energy was more pronounced in the case of hooked fibre and that the hooked fibre also had a more significant positive effect on the tensile and shear strength of the concrete.

With the development of fibre-reinforced concrete, hybrid fibre-reinforced concrete has gradually become a research hotspot. Numerous studies have shown that blending two or more types of fibres can take full advantage of the properties of each fibre material compared to using a single type of fibre [6,7,8,9,10]. This technique can significantly enhance the performance of concrete. Among the types of hybrid fibres, hybrid steel–polypropylene fibre is the most studied and widely used [6,11]. However, because of the large amount of chloride in the marine environment, steel fibres will have the same corrosion problem as reinforcing steel. This was confirmed by Afroughsabet et al. [12] and Frazão et al. [13], who showed that the blending of steel fibres reduced the chloride attack resistance of concrete. Compared with hybrid steel–polypropylene fibre, hybrid basalt–polypropylene fibre is a relatively new form of hybridisation. Compared with steel fibre, basalt fibre (BF) has good corrosion resistance and chemical stability. In addition, BF shows good processability and is less costly [14]. Therefore, hybrid basalt–polypropylene fibre may be a better alternative for marine construction applications.

Owing to the broad application prospects of hybrid basalt–polypropylene fibre, some researchers have conducted a series of studies on hybrid basalt–polypropylene fibre-reinforced concrete (HBPRC). Smarzewski et al. [15,16] investigated the effect of hybrid basalt–polypropylene fibre on the flexural and fracture toughness of concrete. The results showed that the blend of 50% BF and 50% polypropylene fibre (PF) was the optimal combination to improve the flexural toughness of concrete. Furthermore, the hybrid fibre contributed more to improving the fracture energy of concrete compared with a single type of fibre. A study by Shi et al. [17] showed that an appropriate amount of hybrid basalt–polypropylene fibre could significantly improve the flexural strength, post-initial crack strength, and energy absorption property of concrete. Cao [18] reported that hybrid basalt–polypropylene fibre could significantly reduce the dynamic modulus of elasticity, flexural strength, and compressive strength decay rate of concrete under a freeze–thaw environment compared with a single blend of BF or PF. In summary, the current research on HBPRC mainly focuses on the mechanical properties, while few studies have been conducted on the chloride diffusion property. This limits the application and development of HBPRC in the field of marine engineering.

From the perspective of corrosion, the marine environment can be divided into atmospheric, wave splash, tidal, and underwater zones [19]. Among these four regions, concrete structures in the tidal and splash zones are subject to both chloride attack and drying–wetting cycles. During the drying–wetting cycles, corrosive substances will penetrate the concrete at a relatively faster rate under the coupling of multiple complex mechanisms such as diffusion and convection, and the deterioration rate of concrete can reach 3–10 times that of the underwater zone [20,21]. Furthermore, there is a large amount of sulphate in the marine environment. Sulphate does not only react with the hydration products in concrete to form expansive ettringite and gypsum, but also causes the dissolution of calcium ions, which results in the deterioration of concrete properties [22,23]. In addition, the damage pattern and damage mechanism of concrete cannot be considered by a simple superposition of a single erosion factor when both chloride and sulphate are present in the external environment. This is because chloride and sulphate interact and influence each other in a complex manner during the erosion process [24,25,26,27,28]. However, most of the current studies only considered a single chloride environment and neglected the coupling effect of sulphate attack and drying–wetting cycles, which does not correspond to the complexity of the actual marine environment.

In this study, BF and PF were used as reinforcing materials to study the chloride diffusion property of HBPRC subjected to a chloride–sulphate composite solution under drying–wetting cycles. The effects of fibre content, type of fibre, and hybrid form on the chloride content distribution, pore solution pH distribution, and Cl^−^/OH^−^ ratio of concrete at the early and late stages of erosion were analysed. In addition, the composition of the corrosion products and changes in the physical phase components of the concrete were analysed using X-ray powder diffraction (XRD) and thermogravimetric (TG) testing, and the distribution of fibre inside the concrete and corrosion products were observed by scanning electron microscopy (SEM).

## 2. Materials and Methods

### 2.1. Materials and Mix Proportions

**Figure 1:**
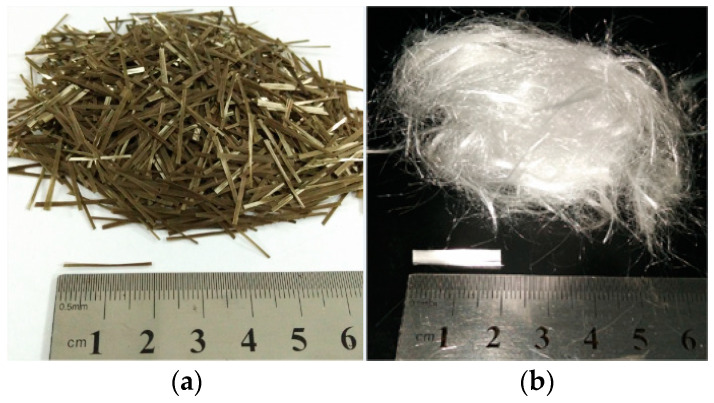
Morphologies of fibres: (**a**) basalt fibre; (**b**) polypropylene fibre.

To prepare the HBPRC, P.O. 42.5R Portland cement (C) (produced by Qinling Cement Xi’an Co., Ltd., Xi’an, Shanxi, China), silica fume (SF) (produced by Dingzhisheng Building Material Co., Ltd., Tianjin, China), fly ash (FA) (produced by Hancheng Datang Shenglong Technology Industrial Co., Ltd., Yulin, Shanxi, China), and ground granulated blast-furnace slag (GGBS) (produced by Lizhilin Building Material Co., Ltd., Yulin, Shanxi, China) were used as cementitious materials. The chemical compositions of the cementitious materials are listed in Table 1. Fine aggregate (S) of river sand with a fineness modulus of 2.8, and coarse aggregates (CAs) of crushed stone with a grain size of 5–20 mm were used. The morphologies of BF (produced by Aerospace Tuoxin Basalt Industry Co., Ltd., Chengdu, Sichuan, China) and PF (produced by Subote New Material Co., Ltd., Nanjing, Jiangsu, China) are shown in Figure 1, and their physical and mechanical properties are presented in Table 2. A polycarboxylic-based superplasticiser (PBS) with a 30% water-reducing rate was used, and potable tap water was utilized for mixing.

The mix proportions of the HBPRC are summarised in Table 3 and the basic mechanical properties of the HBPRC are presented in Table 4, where NC represents concrete without fibre, whereas BC, PC, and BPC denote concretes that are blended with BF, PF, and hybrid BF–PF, respectively. Moreover, 0.1 and 0.2 represent the fibre content (by volume of concrete), and for the hybrid BF–PF, the quantities of BF and PF are equal. For example, BPC-0.1 represents HBPRC with a fibre content of 0.05% by volume for both BF and PF. In addition, the water–cement ratio is 0.38 for all mixtures, and the components are the same except for the fibre dosing.

**Figure 2:**
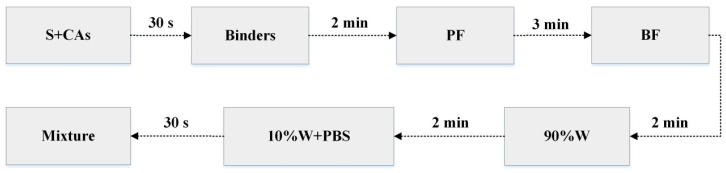
Schematic diagram of the mixing process of HBPRC.

To ensure uniform fibre dispersion, the mixing time of the concrete was extended appropriately, as depicted in Figure 2. After mixing, the mixture was cast in a 100 mm × 100 mm × 100 mm mould and vibrated on a vibrating table for 15 s. The concrete specimens were demoulded after 24 h of casting. Thereafter, the specimens were cured in a standard curing room at a temperature of 20 ± 3 °C and a relative humidity of 95% for 60 days. Finally, the specimens were sealed with epoxy resin before erosion, leaving only one surface for the one-dimensional transport of ions.

### 2.2. Experimental Program

#### 2.2.1. Experimental Procedure

In this study, a composite solution of 3.5% NaCl and 5% Na_2_SO_4_ was used as the erosion solution. The drying–wetting cycle system adopted was as follows: first, the concrete specimens were soaked in the composite solution for 24 h; then, they were dried in an oven at a temperature of 50 °C for 24 h. The solution was changed once a week during the test to maintain a constant ion concentration in the solution. Five erosion periods were set up for the test, namely, drying–wetting cycle for 30 days (15 times), 60 days (30 times), 90 days (45 times), 120 days (60 times), and 180 days (90 times).

#### 2.2.2. Free Chloride Content Test

After each erosion period, one specimen of each group was taken out and placed in a room to dry naturally for 3 days. Then, they were placed in an oven at 50 °C for 24 h. After drying, a concrete grinding machine was used to obtain powder from the surface of the specimen layer by layer. The powder was then passed through a 0.16 mm sieve and then placed inside a sealed bag. The free chloride content of the HBPRC was measured using a potentiometric method, which is in accordance with the Chinese Standard GB/T 50344-2004 [30]. Before testing, the powder was dried in an oven at 50 °C for 24 h and then removed and cooled to room temperature. Subsequently, 5.000 g of the powder was weighed, dissolved in 100 mL of deionised water, shaken in an oscillator for 5 min, left to stand for 24 h, and then filtered. A PXSJ-216F ion meter, a supporting PCl-1 type chloride electrode, and a saturated potassium sulphate reference electrode produced by Shanghai Yidian Scientific instrument Co., Ltd. (Shanghai, China) were used to measure the free chloride content in the filtrate, and the free chloride was calculated using Equation (1).

(1) $$w\;=\;\frac{M\times 10^{-pX}\times V}{G}\times 100\%$$

where w represents the free chloride content, M the molar mass of chloride ion, *pX* the negative logarithm of the molar concentration of chloride in the solution, G the weight of the powder sample, and V the volume of the soaking solution.

#### 2.2.3. Pore Solution pH Test

The pH of the pore solution was also measured using the potentiometric method. The pH test was carried out simultaneously with the free chloride content test, and a standard Pro2100/3C laboratory pH meter produced by Shanghai Yidian Scientific Instrument Co., Ltd. (China) was used to measure the pH of the pore solution for each group from the obtained filtrate.

#### 2.2.4. Microscopic Testing

The XRD test was carried out on the concrete samples using an Empyrean X-ray diffractometer manufactured by Panac (Physical Therapy Association, Shenzhen, Guangdong, China), which has a scan range (2θ) of 5–40°. A Mettler Toledo TGA/DSC 2 thermogravimetric simultaneous differential thermal analyser (Mettler Toledo, Zurich, Switzerland) was used to test the HBPRC after different erosion periods. The mass of the test sample was approximately 15 mg, and the temperature range was 30–900 °C at a heating rate of 10 °C/min (under an N_2_ protective atmosphere). The microstructure of the HBPRC was observed using a Quanta 600 FEG-type cold field emission scanning electron microscope (Carl Zeiss, Jena, Germany) before and after 180 days of erosion.

#### 2.2.5. Theoretical Porosity

The theoretical porosity of the HBPRC can be calculated from the results of the TG test [31], as shown in the following equation:

(2) $$P(\%)\;=\;\frac{V_{por}}{V_{water}+V_{cement}}\times 100\%\;=\;\frac{M_{water}-\frac{BW}{1.3}}{M_{water}+\frac{M_{cement}}{\rho_{cement}}}\times 100\%$$

where *P* represents the theoretical porosity of concrete; $V_{por}$ denotes the pores formed by the evaporation of free water; $V_{water}$ and $V_{cement}$ are the volumes of mixing water and cement, respectively; $M_{water}$ and $M_{cement}$ represent the masses of mixing water and cement, respectively; $\rho_{cement}$ is the density of cement; BW is the mass of chemically bound water, which can be calculated using Equation (3); and the value of 1.3 denotes the average density of the chemically bound water [31].

(3) $$BW(\%)\;=\;\frac{M_{50}-M_{550}}{M_{550}}\times 100\%$$

where $M_{50}$ and $M_{550}$ represent the masses of the sample at 50 °C and 550 °C during the TG test, respectively. It should be noted that Equation (2) is only applicable to the cement paste specimen. For the concrete specimen, Equation (2) can be replaced by Equations (4) and (5).

(4) $$P(\%)\;=\;\frac{V_{por}}{V_{water}+V_{solid}}\times 100\%\;=\;\frac{M_{water}-\frac{BW}{1.3}}{M_{water}+\frac{M_{solid}}{\rho_{solid}}}\times 100\%$$

(5) $$\frac{M_{solid}}{\rho_{solid}}\;=\;\frac{M_{cement}}{\rho_{cement}}+\frac{M_{SF}}{\rho_{SF}}+\frac{M_{FA}}{\rho_{FA}}+\frac{M_{GGBS}}{\rho_{GGBS}}+\frac{M_{S}}{\rho_{S}}+\frac{M_{CAs}}{\rho_{CAs}}$$

where $V_{solid}$ and $M_{solid}$ represent the volume and mass of the solid phase, respectively; $M_{cement}$, $M_{SF}$, $M_{FA}$, $M_{GGBS}$, $M_{S}$, and $M_{CAs}$ denote the masses of cement, SF, FA, GGBS, S, and CAs in concrete, respectively; and $\rho_{cement}$, $\rho_{\mathrm{SF}}$, $\rho_{\mathrm{FA}}$, $\rho_{GGBS}$, $\rho_{S}$, and $\rho_{CAs}$ indicate the densities of cement, SF, FA, GGBS, S, and CAs in concrete, respectively.

## 3. Results and Discussion

### 3.1. Chloride Content

**Figure 3:**
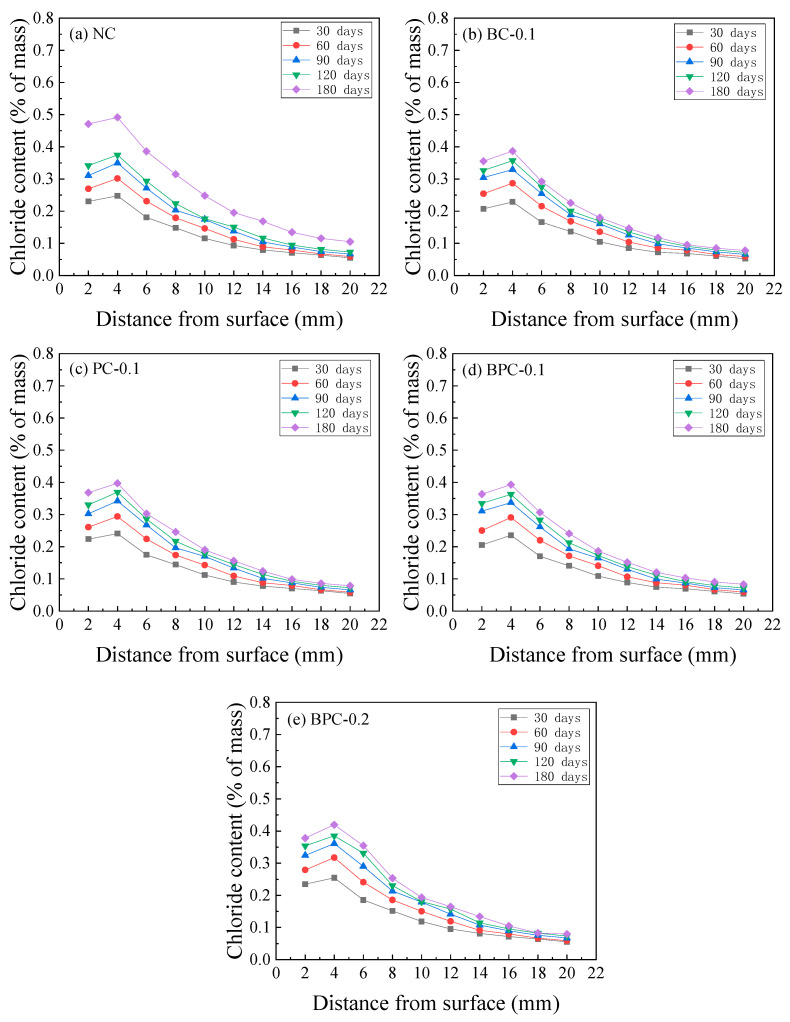
Chloride content distribution of the HBPRC: (**a**) NC; (**b**) BC-0.1; (**c**) PC-0.1; (**d**) BPC-0.1; (**e**) BPC-0.2.

The chloride contents of the HBPRC after different erosion periods are displayed in Figure 3. As indicated, all specimens show a similar chloride content variation pattern, that is, the chloride content increases with erosion time at each depth from the surface of the specimen. In addition, there is a noticeable peak in the distribution curve. Similar results were obtained by Hong et al. [32] and Ye et al. [33]. This is because the pore water in the concrete is not fully saturated when the concrete is under drying–wetting cycles. As a result, the aqueous solution in the surface pores flows, driven by the pore saturation gradients. Hence, the chloride penetration mechanism in the surface layer is a combination of diffusion driven by ion concentration gradients and convection caused by pore saturation gradients. Because the pore structure of concrete has a microstructure similar to that of an “ink bottle–bundle tube”, there will be a lag in the migration of water within the concrete during the drying and wetting processes, which results in an enrichment zone for chloride at a certain depth from the specimen surface. In the area beyond the peak point, chloride will penetrate the concrete by a combination of diffusion and convection in the region called the “convection zone”. Within the peak point, the chloride penetration mechanism is still dominated by diffusion [21]. The width of the convection zone is mainly dependent on factors such as the material of the concrete and the environment where it is exposed. Generally, the depth of the convection zone is approximately 5–20 mm [20,34,35]. It can be observed from Figure 3 that the erosion time and amount of fibre have little effect on the depth of the convection zone, which is approximately 4 mm for each group of specimens after each erosion period.

### 3.2. Effect of Fibre on the Chloride Diffusion Property of Concrete

#### 3.2.1. Early Stage of Erosion

**Figure 4:**
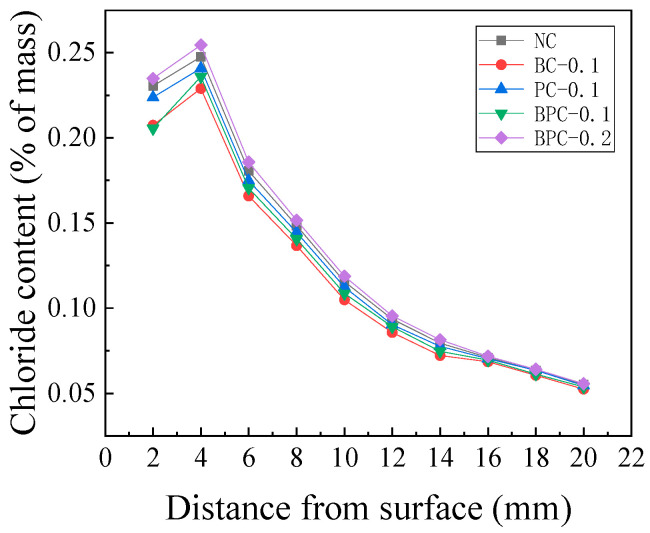
Chloride content distribution of the HBPRC after 30 days drying–wetting cycles.

The chloride content distribution of the HBPRC after 30 days of erosion is depicted in Figure 4. It was found that the chloride penetration rate inside the concrete at the early stage of erosion could be reduced by blending fibres with a volume fraction of 0.1%; however, this reduction was not very remarkable and the magnitude of the reduction was affected by the type of fibre. The reduction effect of BF on the chloride penetration rate was greater than that of PF, while that of the hybrid BF–PF was in between. Taking the chloride content of the peak point as an example, the peak point chloride content of specimens BC-0.1, PC-0.1, and BPC-0.1 decreased by 7.9, 2.7, and 4.7%, respectively, compared with the control specimen NC. However, the peak point chloride content of specimen BPC-0.2 was 2.8% higher than that of specimen NC after 30 days of erosion. This result indicates that excessive blending of fibre increases the chloride penetration rate inside the concrete during the early stage of erosion.

The main reason that an appropriate amount of fibre can reduce the initial chloride penetration rate is that the fibre can effectively prevent the formation and development of shrinkage microcracks during the hardening process of concrete [36,37,38]. This is because the presence of microcracks significantly increases the chloride penetration rate into the concrete. Boulfiza et al. [39] compared the effective permeability of cracked and uncracked concretes and found that the effective permeability of the cracked matrix was 13 orders of magnitude higher than that of the uncracked matrix. Meanwhile, because the internal structure of concrete is not significantly affected by erosion at the early stage, the resistance of concrete to chloride attack at the early stage is mainly influenced by the performance of the matrix at the end of curing. Therefore, appropriate amounts of BF, PF, and hybrid BF–PF can reduce the chloride penetration rate at the early stage of erosion.

However, a large amount of paste is often required to wrap the fibre in the concrete mixing process. This is because of the large specific surface area of the fibre, in which a layer called the interfacial transition zone (ITZ) with high porosity will form on the fibre surface. The ITZ is the weakest area in the composite system and contains a large number of connected pores [40,41]. Although the permeability and porosity of concrete are not synonymous, it is generally accepted that the permeability of concrete decreases with increasing porosity [37]. Therefore, the ITZ with high porosity weakens the effect of fibre bridging on the chloride resistance of concrete to some extent. The property of the fibre-matrix ITZ is mainly influenced by fibre wettability and the fibre “wall effect” [36,38]. It is generally believed that the better the wettability of the fibre and the weaker the wall effect, the better the performance of the fibre-matrix ITZ and the lower the porosity. BF is hydrophilic, and the BF adopted in this study is only 15 μm in diameter; thus, its wall effect on the solid particles of the HBPRC is relatively insignificant. Hence, BF has a good bonding property with the matrix, and the BF-matrix ITZ is thin and has low porosity. In contrast, PF is hydrophobic [40] and has a larger diameter (30 μm) than BF, which leads to a stronger wall effect. As a result, PF has a poorer bonding property with the matrix and a wider ITZ with higher porosity compared to BF [40]. This may be the main reason that PF is less effective than BF in improving the chloride attack resistance of concrete at the early stage of erosion.

When the amount of hybrid BF–PF reaches 0.2%, the excessive fibre blending reduces the dispersion of fibre. Consequently, the fibre tends to clump and entangle; this introduces a large number of air bubbles, resulting in numerous air voids in the hardened concrete. In addition, a large number of fibre-matrix ITZ will change the original pore structure of the concrete, making the pores interconnected along the fibre length direction. Therefore, the incorporation of excess fibre in specimen BPC-0.2 provides more channels for ions to enter the concrete, thus increasing the chloride penetration rate in concrete at the early stage of erosion.

#### 3.2.2. Late Stage of Erosion

**Figure 5:**
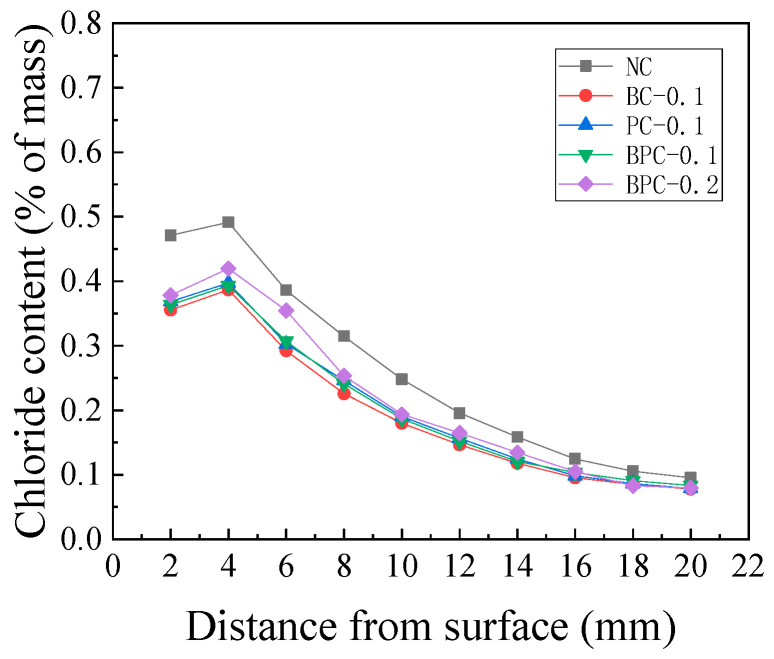
Chloride content distribution of the HBPRC after 180 days drying–wetting cycles.

**Figure 6:**
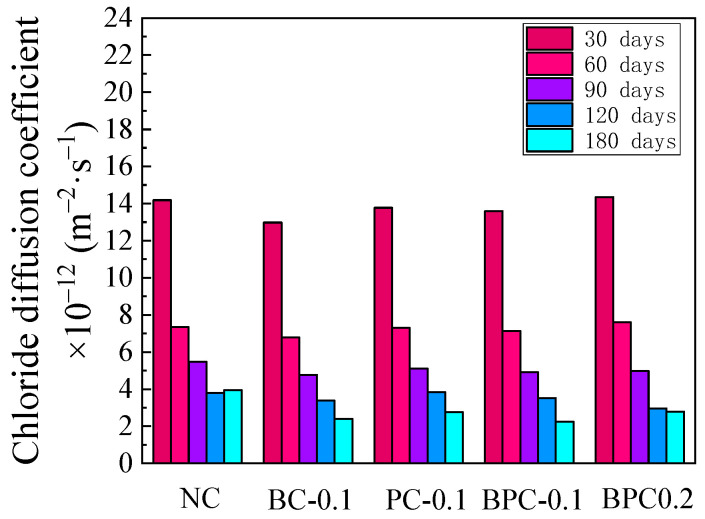
Apparent chloride diffusion coefficient.

The chloride content distribution of the HBPRC after 180 days of erosion is illustrated in Figure 5. As shown, the positive effect of the fibre on the resistance of concrete to chloride attack becomes evident after a longer period of erosion. After 180 days of erosion, the peak point chloride contents of specimens BC-0.1, PC-0.1, BPC-0.1, and BPC-0.2 decreased by 24.3, 21.5, 20.5, and 8.1%, respectively, compared to that of the control specimen NC. It is obvious that the reductions are significantly higher than those after 30 days of erosion, and even the chloride-content peak point of specimen BPC-0.2 is lower than that of specimen NC, which is notably different from the situation after 30 days of erosion. Therefore, the variation pattern of the chloride content of the HBPRC during 30–180 days of erosion needs to be further analysed. As described in Section 3.1, the chloride penetration mechanism within the convection zone is still dominated by diffusion. Therefore, the penetration process of chloride within the convection zone can be approximately described by Fick’s second law, which is expressed as.

(6) $$C(x,t)\;=\;C_{0}+(C_{s}-C_{0})(1-erf\frac{x}{2\sqrt{Dt}})$$

where C(x,t)  represents the chloride content, $C_{s}$ the surface chloride content, *D* the apparent chloride diffusion coefficient, x the depth, and t the corrosion time. The apparent chloride diffusion coefficient of the HBPRC, calculated using Equation (6), is presented in Figure 6. It should be noted that only the chloride content data up to the peak point were used. Figure 6 shows that the apparent chloride diffusion coefficients of all specimens decrease with increasing erosion time between 30 and 120 days of erosion. There are two possible reasons for this result. First, with the continuous hydration of the cementitious material, the concrete is gradually compacted, and the property of the fibre-matrix ITZ is improved. Second, as shown in Equations (7)–(9), the sulphate in the composite solution generates ettringite and gypsum with hydration products [41], which fill the pores and the fibre-matrix ITZ. Although the presence of sulphate reduces the stability of Friedel’s salt, thus releasing part of the bound chloride [42], the inhibition of chloride penetration due to the porosity reduction is much greater. Therefore, the apparent chloride diffusion coefficient of concrete continues to decrease.

(7) $$Na_{2}SO_{4}\cdot \;10H_{2}O+Ca{\left(OH\right)}_{2}\to CaSO_{4}\cdot \;2H_{2}O+2NaOH+8H_{2}O$$

(8) $$3(CaSO_{4}\cdot \;2H_{2}O)+3CaO\cdot \;Al_{2}O_{3}+26H_{2}O\to 3CaO\cdot \;Al_{2}O_{3}\cdot \;CaSO_{4}\cdot \;32H_{2}O$$

(9) $$Ca{\left(OH\right)}_{2}+Na_{2}SO_{4}+2H_{2}O\to CaSO_{4}\cdot \;2H_{2}O+2NaOH$$

Figure 6 indicates that the apparent chloride diffusion coefficient of specimen NC shows an upward trend after 120 days of erosion. This may be caused by cracking due to the corrosion products, as the expansive ettringite and gypsum generate local expansion stress on the walls of the pores. When the stress exceeds the tensile strength of the concrete matrix, microcracks form and gradually develop into macrocracks. Compared with the filling effect of the hydration products and corrosion products on the pores, the accelerating effect of cracks on chloride penetration is more significant. Thus, the apparent chloride diffusion coefficient of specimen NC increased during the late stage of erosion. This agrees well with Jin’s study [43], whose result also indicated that sulphate decreased the chloride penetration rate in the short term but increased it in the long term. However, the apparent chloride diffusion coefficient of the specimens with fibres continued to decrease after 120 days of erosion. This is because the fibre can effectively prevent the formation and development of sulphate corrosion microcracks, thus reducing the acceleration effect of sulphate corrosion microcracks on chloride penetration at the late stage of erosion.

Specimen BPC-0.1 exhibited the lowest apparent chloride diffusion coefficient after 180 days of erosion, which was 43.1% lower than that of specimen NC. Although the variability in the experimental results cannot be excluded, these results are also explainable. According to El-Hamrawy’s study, PF with a low elastic modulus can bridge microcracks and delay the development of microcracks into macrocracks, while BF with a high elastic modulus can inhibit the expansion of macrocracks [7]. As a result, BF and PF form a complex fibre network, which can prevent the formation and development of sulphate attack microcracks more effectively than BF or PF alone.

From Figure 6 and the above analysis, it can be found that the relationship between chloride diffusion coefficient and erosion time of each group of specimens can be approximately described by quadratic polynomial function, namely *D* = A + B*t* + C*t*^2^, where *t* is the erosion time (days); A, B, and C are material property parameters determined from tests. It is obvious that the larger A is, the worse the concrete’s resistance to chloride attack at the early stages of erosion; the smaller B and the larger C are, the earlier sulphate attack begins to have an adverse effect on the chloride attack resistance of concrete. The regression analysis is carried out according to the experimental data, and the results are shown in Table 5. Table 5 indicates that there is a good correlation between the chloride diffusion coefficient and erosion time for all groups of specimens, with correlation coefficients exceeding 0.96. Specimen BC-0.1 had the smallest A. In addition, the incorporation of fibres increased B and decreased C, and specimen BPC-0.1 had the largest B and the smallest C, which increased by 8.99% and decreased by 18.16%, respectively, compared to specimen NC. This indicates that the 0.1% hybrid BF–PF was the most effective in inhibiting sulphate erosion, which is consistent with the results of the above analysis.

### 3.3. Pore Solution pH Distribution

**Figure 7:**
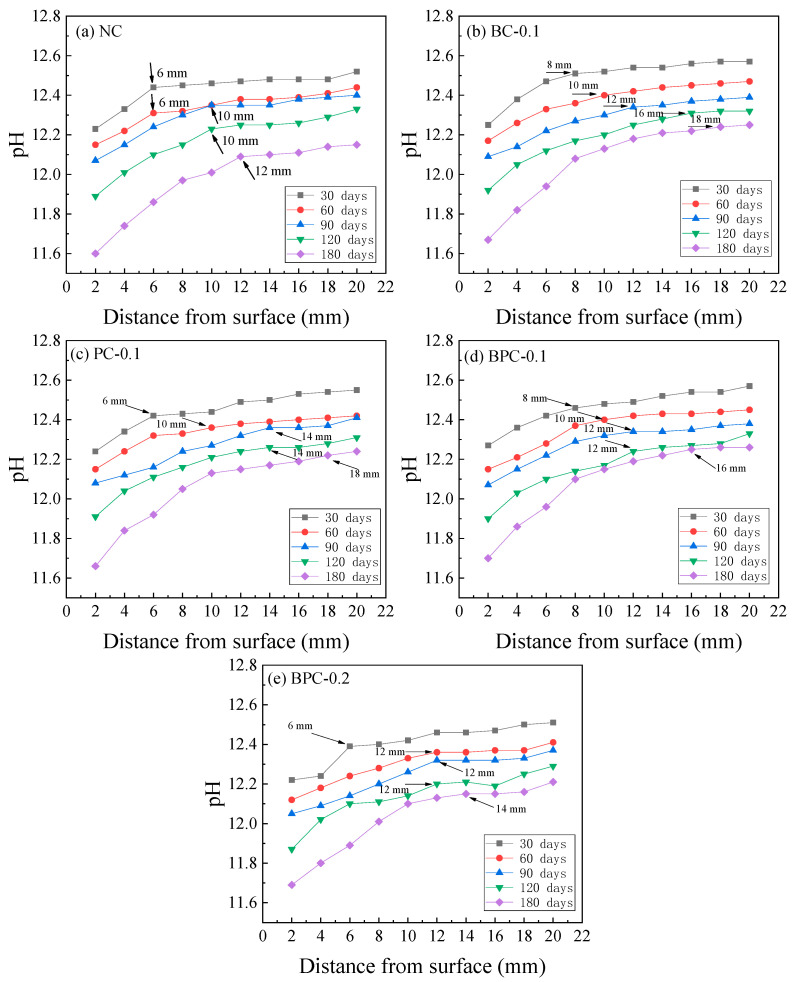
pH distribution of the HBPRC: (**a**) NC; (**b**) BC-0.1; (**c**) PC-0.1; (**d**) BPC-0.1; (**e**) BPC-0.2.

As indicated in Equations (7) and (9), the sulphate attack consumes Ca^2+^ in the concrete pore solution and generates readily soluble NaOH. Because the pH of the erosion solution is much lower than that in the concrete pore solution, NaOH gradually diffuses to the outside. This leads to a gradual decrease in the alkalinity of the concrete pore solution, which seriously affects the stability of the concrete matrix [44]. The pore solution pH distributions of the HBPRC after different erosion periods are depicted in Figure 7. It can be observed that the pore solution pH decreases with increasing erosion time for all groups of specimens, and the closer the location to the specimen surface, the more evident the decrease. In addition, as shown by the arrows in Figure 7, with the increase in erosion time, the area with a large pH change gradually moves to the interior of the concrete, which indicates that the influence depth of the sulphate attack gradually increases. However, because the penetration rate of sulphate in concrete is much slower than that of chloride [28,45], the development of the sulphate attack depth is relatively slow.

Similar to the difference in the degree of influence of the fibre on the chloride diffusion property at the early and late stages of erosion, the influence of the fibre on the degree of pH reduction of the concrete pore solution also differs. The fibre had minor beneficial effect on inhibiting the pH reduction at the early stage of erosion and the incorporation of 0.2% hybrid BF–PF even slightly accelerated the pH reduction. Take the pore solution pH at 4 mm from the surface of the specimen as an example. After 30 days of erosion, the pore solution pH values of specimens BC-0.1, PC-0.1, and BPC-0.1 are only 0.01–0.05 higher than that of specimen NC, whereas that of specimen BPC-0.2 is 0.09 lower. In contrast, after 180 days of erosion, the pore solution pH values of the specimens with fibres are all significantly higher than that of specimen NC, and the pore solution pH values of specimens BC-0.1, PC-0.1, BPC-0.1, and BPC-0.2 are approximately 0.06–0.12 higher than that of specimen NC. A higher liquid phase alkalinity is the basis for maintaining a stable concrete system. When the liquid phase alkalinity decreases, Ca(OH)_2_(CH) in the solid phase of concrete will dissolve to maintain the alkalinity of the concrete pore solution. While CH is not only the basis for the stable presence of hydration products such as C–S–H gels, it is also an important component of the hardened cement paste itself [37]. Therefore, when CH and C–S–H gels continue to decompose and diffuse to the outside as sulphate continues to enter the concrete interior, the properties of the concrete matrix will continue to deteriorate. Thus, it can be concluded that blending an appropriate amount of hybrid BF–PF into concrete can both improve the chloride attack resistance of concrete and retard the deterioration rate of the concrete matrix properties.

**Figure 8:**
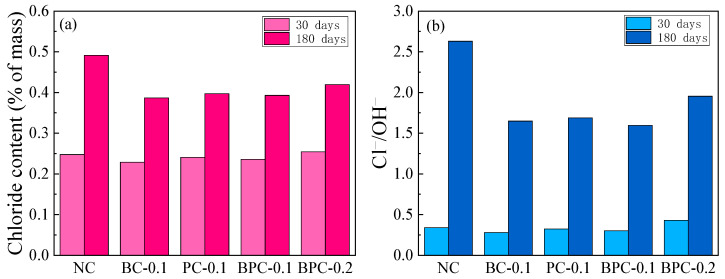
The chloride content and Cl^−^/OH^−^ ratio of the HBPRC after 30 days and 180 days drying–wetting cycles: (**a**) chloride content; (**b**) Cl^−^/OH^−^ ratio.

In the case of reinforced concrete structures, the decrease in alkalinity of the concrete pore solution also decreases the stability of the reinforcement passivation film, thus increasing the risk of reinforcement corrosion. It is well known that the molar ratio of free chlorides to hydroxide ions (Cl^−^/OH^−^) can be used to assess the corrosion potential of reinforcing steel in a chloride-containing environment [24]. In this context, it is clear that the blending of fibre has an effect on the variation in both chloride and hydroxide contents during erosion. For this reason, a comparative analysis of the influence of fibre on the variation in chloride content and Cl^−^/OH^−^ ratio was performed. Similarly, taking the location of 4 mm from the surface as an example, the chloride contents and Cl^−^/OH^−^ ratios of the HBPRC after 30 days and 180 days of erosion are illustrated in Figure 8. As shown, the growth rate of Cl^−^/OH^−^ ratio is much higher than that of the chloride content for all specimens. For example, the Cl^−^/OH^−^ ratio of specimen NC after 180 days of erosion is 7.7 times higher than that after 30 days of erosion, while the chloride content increases by only approximately two times. This result shows that sulphate severely affects the risk of reinforcement corrosion. First, it causes cracking of the concrete matrix, which increases the rate of chloride penetration. Second, it decreases the alkalinity of the concrete pore solution, which further increases the risk of reinforcement corrosion.

Figure 8b also shows that the Cl^−^/OH^−^ ratios of the specimens with fibres are much smaller than that of specimen NC at the late stage of erosion. After 180 days of erosion, the Cl^−^/OH^−^ ratios of specimens BC-0.1, PC-0.1, BPC-0.1, and BPC-0.2 are reduced by 37.5%, 35.8%, 39.3%, and 25.7%, respectively, compared to that of specimen NC. This is mainly because the fibre prevents the formation and development of sulphate attack microcracks, which reduces the chloride penetration rate inside the concrete as well as the penetration rate of sulphate. In addition, similar to the apparent chloride diffusion coefficient of the HBPRC after 180 days of erosion, specimen BPC-0.1 also exhibits the smallest Cl^−^/OH^−^ ratio after 180 days of erosion, which further demonstrates the superiority of the hybrid BF–PF over BF or PF alone in improving the long-term durability of concrete in marine environments.

### 3.4. XRD Analysis

**Figure 9:**
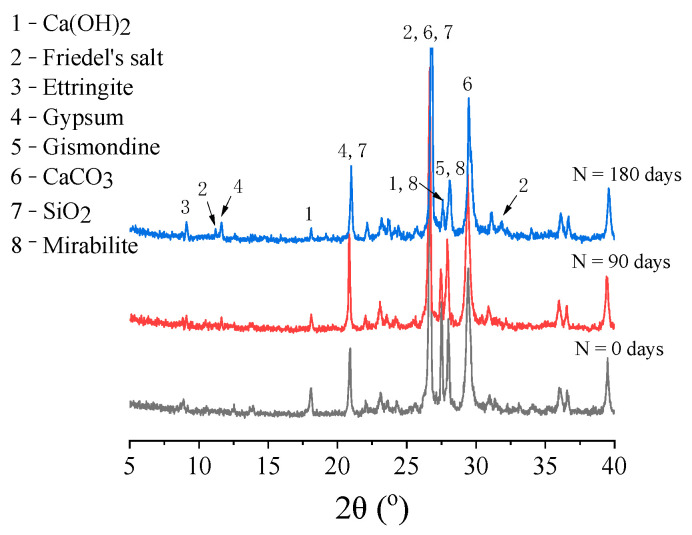
X-ray powder diffraction (XRD) patterns of specimen NC.

The XRD patterns of specimen NC after different erosion periods are displayed in Figure 9. As shown, the crystalline phases of the HBPRC are mainly Ca(OH)_2_(CH), Friedel’s salt, ettringite, gypsum, gismondine, CaCO_3_, SiO_2_, and mirabilite. In this study, the focus is on the transformation of the hydration products and the variations in the Friedel’s salt, ettringite, and gypsum.

It can be seen from Figure 9 that there is a Friedel’s salt diffraction peak (11.25° 2θ) in the specimen after 180 days of corrosion. This indicates that chloride had entered the concrete and would be bound in the concrete in the form of Friedel’s salt. However, the diffraction peak of Friedel’s salt is not remarkable at 90 days of erosion. This is because the sulphate in the composite solution preferentially reacts with the aluminium phase (SO_4_–AFm, C_4_AF, and C_3_A) in the hydration products to form ettringite, which is much more stable than Friedel’s salt [26,46]. Therefore, owing to the consumption of a large amount of aluminium phase, the chloride binding capacity of concrete is reduced. However, because the chloride penetration rate in concrete is faster than that of sulphate [47], the increase rate of chloride is much higher than that of sulphate. Although ettringite is more stable than Friedel’s salt, both necessarily follow thermodynamic equilibrium conditions (the thermodynamic equilibrium constants of Friedel’s salt and ettringite are presented in Table 6). Hence, when the chloride content in the pore solution is much higher, the stability of ettringite decreases while the stability of Friedel’s salt increases, which explains the appearance of a more pronounced Friedel’s salt diffraction peak after 180 days of erosion.

The ettringite diffraction peak (9.08° 2θ) and gypsum diffraction peak (11.54° 2θ) are also observed in Figure 9, and the intensities of the ettringite and gypsum diffraction peaks gradually increase with increasing erosion time. This shows that ettringite and gypsum in the concrete increase gradually with increasing erosion time. As the resulting ettringite and gypsum gradually filled the concrete pores and reduced the channels for chloride penetration, the apparent chloride diffusion coefficient of concrete decreased continuously between 30 and 120 days of erosion. Furthermore, it can be observed from Figure 9 that the intensity of the CH diffraction peak decreases with increasing erosion time, which verifies the previous statement. It should be noted that sodium sulphate gradually crystallises and precipitates in the pores owing to the continuous supply of sodium sulphate and the drying–wetting cycles. Consequently, a diffraction peak of mirabilite ($Na_{2}SO_{4}\cdot \;10H_{2}O$) is also observed in the XRD pattern, which indicates that the physical erosion of sulphate may also be a cause of cracking of the concrete matrix.

### 3.5. TG Analysis

**Figure 10:**
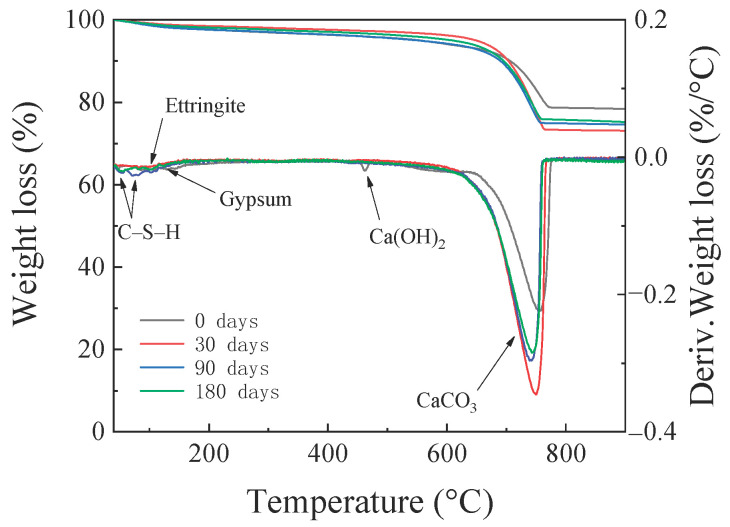
Thermogravimetric (TG) analysis of the HBPRC.

The TG and derivative thermogravimetric (DTG) curves of specimen NC after different erosion periods are depicted in Figure 10. Three weight loss peaks can be clearly observed in the DTG curve. The weight loss peak at 50–80 °C is mainly due to the evaporation of bound and free water from the hydration products (e.g., C–S–H gels) [50]. The second weight loss peak is located at 420–460 °C, which corresponds to the decomposition of CH. It can be observed from Figure 10 that the CH weight loss peak almost disappears after 30 days of erosion, which implies that the CH has been consumed in large amounts. The weight loss peak at 750–850 °C corresponds to the decomposition of calcium carbonate. In addition to these three obvious weight loss peaks, after 180 days of erosion, there are weight loss peaks at 90–110 °C and 130–140 °C, which correspond to the heat uptake peaks of ettringite and gypsum dehydration, respectively [51]. Moreover, as described in Section 3.4, the presence of sulphate reduces the stability of Friedel’s salt. Therefore, the weight loss peak of Friedel’s salt (heat absorption peaks for the evaporation of free water in the intercalated space between 120 and 140 °C and heat absorption peaks for the weight loss from dehydroxylation between 286 and 322 °C) is not significant. Generally, the results of the TG/DTG analysis are in good agreement with those of the XRD analysis.

**Figure 11:**
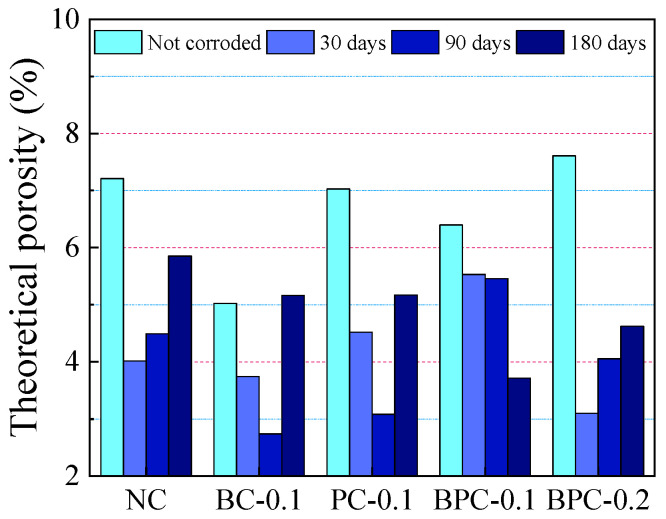
Theoretical porosity variation at 4 mm from the surface of the HBPRC.

**Figure 12:**
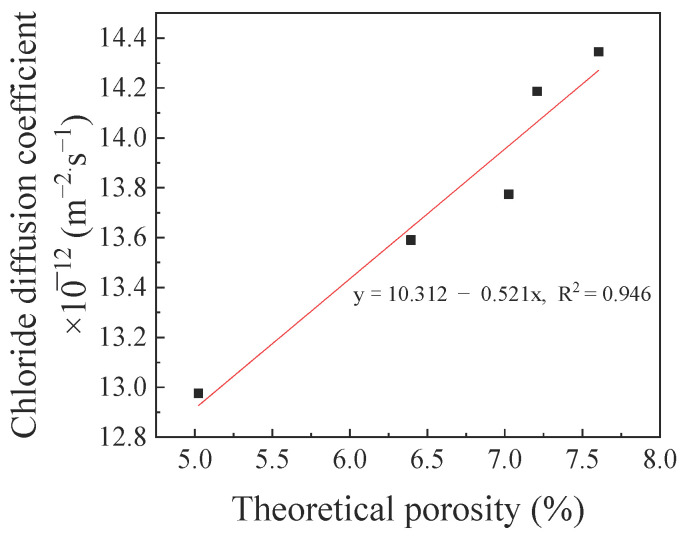
The relationship between chloride diffusion coefficient and theoretical porosity.

The porosity is an important factor that affects the chloride diffusion performance of concrete. The variations in the theoretical porosity of the HBPRC were calculated based on Equations (3)–(5), and the results are presented in Figure 11. This method provides neither the absolute pore volume nor the pore size distribution but it can be used as an evaluation method [31,50]. Figure 11 shows that the blending of BF, PF, and hybrid BF–PF reduces the initial porosity of the concrete when the amount of fibre is 0.1%. Compared to the porosity of specimen NC, the initial porosities of specimens BC-0.1, PC-0.1, and BPC-0.1 are reduced by 30.31, 2.56, and 11.29%, respectively. However, when the amount of hybrid BF–PF increases to 0.2%, its porosity increases by 5.51% compared with that of specimen NC. The initial porosity of concrete is closely related to the chloride penetration rate at the early stage of erosion. Figure 12 displays the relationship between the initial porosity of the HBPRC and the apparent chloride diffusion coefficient during the first 30 days of erosion. It can be observed that there is a linear relationship between the two parameters, and the correlation is good. The apparent chloride diffusion coefficient decreases with decreasing initial porosity.

Compared with the initial porosity, the porosity of the HBPRC decreases by 13.49–59.24% after 30 days of erosion. However, it should be noted that the change in porosity is not only caused by the corrosion products filling the pores. According to Haga et al. [52], the dissolution of CH in concrete would lead to an increase in the pore volume with a diameter of a few micrometres, while the dissolution of C–S–H would lead to an increase in the pore volume with a diameter of less than 100 nm. Additionally, microcracks generated by excess corrosion products increase the porosity of concrete [53]. Therefore, the change in porosity is the result of the combination of these three factors, that is, when the pore volume reduced by the corrosion products ($\delta V^{-}$) is greater than that increased by the CH and C–S–H dissolution and corrosion-generated microcracks ($\delta V^{+}$), the porosity decreases; when $\delta V^{-}$ is smaller than $\delta V^{+}$, the porosity of concrete increases. This shows that the change in porosity is dominated by $\delta V^{-}$ during the first 30 days of erosion. However, after 30 days of erosion, the change trend in the porosity of each group differs. As can be seen from Figure 11, the porosity of specimen NC decreases to its lowest value after 30 days of erosion. Then, the porosity increases rapidly, implying a rapid increase in the corrosion of specimen NC, with $\delta V^{+}$ beginning to dominate the porosity change. This result clearly explains the increasing trend of the chloride diffusion rate in specimen NC at the late stage of erosion. Compared with specimen NC, the addition of 0.1% BF, PF, and hybrid BF–PF delayed the corrosion age at which the concrete porosity started to increase. The porosities of specimens BC-0.1 and PC-0.1 started to increase after 90 days of erosion, while that of specimen BPC-0.1 did not show a decreasing trend until 180 days of erosion. However, in contrast to the specimens with 0.1% fibre blending, the 0.2% hybrid BF–PF was not effective in retarding the age of erosion at which porosity starts to increase. Similar to specimen NC, the porosity of specimen BPC-0.2 started to increase after 30 days of erosion. This is probably because excessive fibre incorporation reduces the dispersibility of the fibre, which leads to easy clumping and folding of the fibre, thereby preventing full utilisation of its function.

### 3.6. SEM Analysis

**Figure 13:**
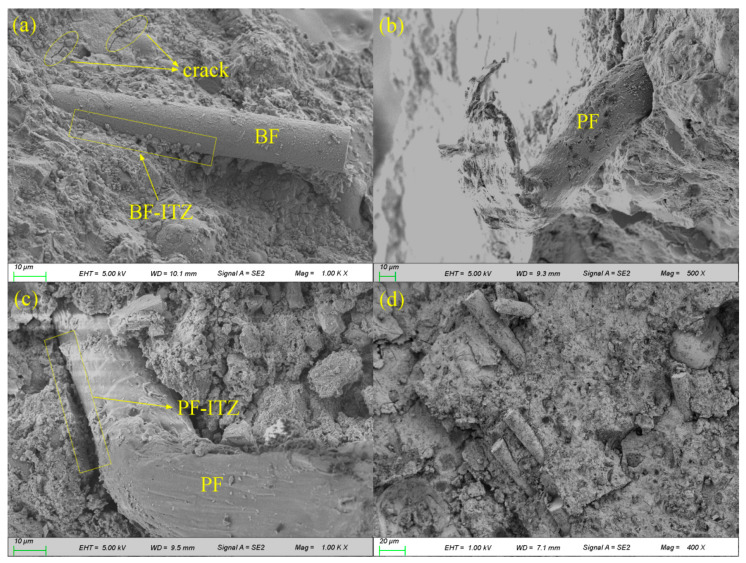
Morphology of BF and PF in the HBPRC before erosion: (**a**) BC-0.1; (**b**) and (**c**) PC-0.1; (**d**) BPC-0.2.

To better understand the effects of the hybrid BF–PF and sulphate on the resistance of concrete to chloride attack, the microscopic morphologies of the HBPRC before and after erosion were examined by SEM. The microscopic morphology of the HBPRC before erosion is depicted in Figure 13. As shown in Figure 13a, there are several microcracks with small diameters near the BF that did not expand owing to the presence of BF. This means that BF can effectively prevent the formation and development of shrinkage microcracks during the hardening stage of concrete. In addition, Figure 13a shows that the BF is tightly bonded to the concrete matrix with a thin and low-porosity fibre-matrix ITZ. Figure 13b presents an SEM image of the end buckling of PF after pulling off. During this process, a large amount of fracture energy could be consumed. Thus, PF can also effectively inhibit the extension of microcracks. Figure 13c shows that only a small amount of cement paste adhered to the surface of the PF after it was pulled out. This means that the bonding performance of PF to the concrete matrix is poor. Therefore, a wider and more porous PF-matrix ITZ is observed. The difference between the PF-matrix ITZ and BF-matrix ITZ leads to the difference in the improvement in the chloride attack resistance of concrete at the early stage of erosion, as previously described. Figure 13d reveals the presence of inhomogeneous fibre dispersion in specimen BPC-0.2, which reduced the bridging effect of the fibre, and the mutual overlap of the fibre-matrix ITZ produced more serious defects. Therefore, the blending of 0.2% hybrid BF–PF reduces the chloride attack resistance of concrete at the early stage of erosion.

**Figure 14:**
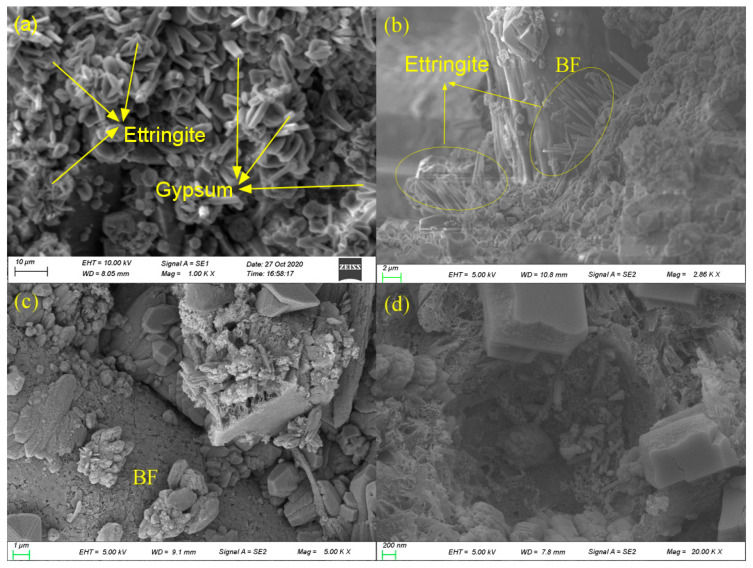
SEM micrographs of specimen BC-0.1 after 180 days of erosion: (**a**) corrosion products; (**b**) and (**c**) BF-ITZ; (**d**) pore with corrosion products.

The microscopic morphology of the HBPRC after 180 days of erosion is displayed in Figure 14. Figure 14a reveals the presence of a large amount of columnar gypsum and needle-like ettringite in the HBPRC after 180 days of erosion, which is in accordance with the XRD and TG results. Figure 14b,c show that a large amount of corrosion products filled the fibre-matrix ITZ, and the needle-like ettringite provided an anchoring effect between the fibre and matrix, which improved the mechanical bite force between the fibre and the matrix. At the same time, the corrosion products also decreased the porosity of the fibre-matrix ITZ, thus reducing the adverse effect of the fibre-matrix ITZ on the chloride attack resistance of concrete. Figure 14d indicates that a large amount of corrosion products filled the pores inside the concrete. Owing to the filling effect of corrosion products on both the fibre-matrix ITZ and the pores, the porosity of concrete decreased significantly during the erosion process; hence, the apparent chloride diffusion coefficient of the HBPRC gradually decreased in the short term.

## 4. Conclusions

The purpose of this study was to evaluate the effect of hybrid basalt–polypropylene fibre on the chloride diffusion property of concrete subjected to a chloride–sulphate composite solution under drying–wetting cycles. Based on the results, the following conclusions can be drawn:

1. There is a peak at a certain depth from the specimen surface in the chloride content distribution curve due to drying–wetting cycles and the “ink bottle–bundle tube” like microstructure of concrete pores; however, the fibre and erosion time have little effect on the depth of the peak point. The peak depth was approximately 4 mm in each group of specimens after each erosion period.
2. When the fibre content was 0.1%, the addition of BF, PF, and hybrid BF–PF improved the chloride attack resistance of concrete at the early stage of erosion. Adding 0.1% BF had the best effect, which reduced the apparent chloride diffusion coefficient by 8.5% after 30 days of erosion. Moreover, the chloride attack resistance of concrete at the early stage of erosion was reduced when 0.2% hybrid BF–PF was blended.
3. The sulphate in the composite solution decreased and increased the chloride penetration rate in the concrete at the early and late stages of erosion, respectively. In addition, sulphate erosion also led to a decrease in the alkalinity of the pore liquid phase of concrete, in which the growth rate of the Cl^−^/OH^−^ ratio was 2.8 to 3.9 times the growth rate of the chloride content.
4. The positive effect of the fibre gradually appeared as the erosion time was extended. After 180 days of erosion, the apparent chloride diffusion coefficients of the specimens with fibre were reduced by 29.4–43.1% compared with that of the control specimen NC, while the Cl^−^/OH^−^ ratios were reduced by 25.7–39.3%. The specimen blended with 0.1% hybrid BF–PF demonstrated the smallest apparent chloride diffusion coefficient and Cl^−^/OH^−^ ratio at the late stage of erosion.
5. The theoretical porosity of concrete decreases with the addition of fibre; however, an excessive amount of fibre increases the porosity. The SEM results show that BF has a better bonding performance with the concrete matrix and a thinner fibre-matrix ITZ with lower porosity compared with PF. The corrosion products filled the pores inside the concrete as well as the fibre-matrix ITZ during the erosion process, thus reducing the porosity of the concrete.

From the above conclusions, it can be found that fibres have little effect on the chloride diffusion properties of concrete in a chloride–sulphate composite solution under drying–wetting cycles at the early stages of erosion. Its effect is mainly reflected when the expansion products begin to produce expansion stress on the pore wall, which is mainly due to its anti-cracking effect. However, it is also clear from the above conclusions that an excessive amount of fibre increases the porosity of the concrete. There are two main reasons for this: on the one hand, the high porosity of the fibre-matrix ITZ; on the other hand, excessive use of fibre reduces the dispersion of the fibre, resulting in the introduction of air bubbles in the mixing process. For a single chloride attack environment, the increase of porosity reduces the chloride attack resistance of the concrete, because it provides more channels for chloride penetration. However, this effect may change for a chloride-sulphate composite environment. The matrix with high porosity can provide more space to accommodate sulphate erosion products, which can also inhibit the formation of sulphate erosion cracks, which is described by other scholars as the buffer capacity of pores. This mechanism may also play a very important role in the process of erosion resistance. Of course, high porosity will inevitably provide more channels for chloride penetration, and further research is needed to confirm the optimum mix ratio for a good balance between the two.

## Figures and Tables

**Table 1:** Chemical composition of binders [29].

Composition (wt. %)	CaO	SiO_2_	Al_2_O_3_	Fe_2_O_3_	MgO	SO_3_	Other
Cement	63.42	21.18	5.02	3.14	3.12	2.3	1.82
SF	1.63	85.04	0.97	1.04	0.32	-	10
FA	21.14	35.71	16.57	8.92	1.41	1.94	12.49
GGBS	34.11	34.65	14.21	0.49	11.15	1	3.74

SF, silica fume; FA, fly ash; GGBS, ground granulated blast-furnace slag.

**Table 2:** Physical and mechanical properties of BF and PF.

Type	Length (mm)	Diameter (μm)	Aspect Ratio	Density (kg/m^3^)	Elastic Modulus (MPa)	Tensile Strength (MPa)	Elongation (%)
BF	18	15	1200	2560	75,000	4500	3.15
PF	19	30	633	910	3000	270	40

BF, basalt fibre; PF, polypropylene fibre.

**Table 3:** Mix proportions of the HBPRC (kg/m^3^).

Mixture	Binder				PBS	W	S	CAs	BF	PF
	Cement	SF	FA	GGBS						
NC	241.6	15.8	79.2	59.4	3.96	150.5	683.4	1163.6	0	0
BC-0.1	241.6	15.8	79.2	59.4	3.96	150.5	683.4	1163.6	2.56	0
PC-0.1	241.6	15.8	79.2	59.4	3.96	150.5	683.4	1163.6	0	0.91
BPC-0.1	241.6	15.8	79.2	59.4	3.96	150.5	683.4	1163.6	1.28	0.46
BPC-0.2	241.6	15.8	79.2	59.4	3.96	150.5	683.4	1163.6	2.56	0.91

HBPRC, hybrid basalt–polypropylene fibre-reinforced concrete; S, fine aggregate; CAs, coarse aggregate; W, water; PBS, polycarboxylic-based superplasticiser.

**Table 4:** Mechanical properties of the HBPRC.

Mixture	Compressive Strength (MPa)				Split Tensile Strength (MPa)				Modulus of Elasticity (GPa)			
	7 days	14 days	28 days	60 days	7 days	14 days	28 days	60 days	7 days	14 days	28 days	60 days
NC	28.09	35.33	42.29	45.70	2.75	3.19	3.66	3.80	26.8	30.3	34.1	36.4
BC-0.1	32.00	40.12	46.68	49.42	3.22	3.61	4.01	4.10	26.3	31.0	34.5	35.5
PC-0.1	28.74	36.14	43.10	45.77	3.12	3.49	3.95	4.06	26.5	30.9	34.2	35.6
BPC-0.1	30.05	37.93	44.43	47.23	3.03	3.37	3.80	4.02	27.1	30.3	34.0	35.7
BPC-0.2	26.41	33.47	40.81	43.98	3.07	3.31	3.94	3.90	26.8	30.2	34.1	36.1

**Table 5:** Fitting results of material characteristic parameters.

Mixture	A	B	C	R^2^
NC	20.11241	−0.2435	8.59 × 10^−4^	0.9683
BC-0.1	18.14847	−0.2216	7.21 × 10^−4^	0.9704
PC-0.1	19.19045	−0.2233	7.40 × 10^−4^	0.9692
BPC-0.1	18.98268	−0.2129	7.03 × 10^−4^	0.9715
BPC-0.2	20.83186	−0.23768	8.49 × 10^−4^	0.9857

**Table 6:** Thermodynamic equilibrium constant of ettringite and Friedel’s salt at 25 °C.

Mineral	Reaction	Calculated log K_sp_	Ref.
Ettringite	K_sp_ = (Ca^2+^)^6^·(Al${\left(OH\right)}_{4}^{-}$)^2^·(${SO}_{4}^{2-}$)^3^·(OH^−^)^4^	−44.00	[48]
Friedel’s salt	K_sp_ = (Ca^2+^)^4^·(Al${\left(OH\right)}_{4}^{-}$)^2^·(Cl^−^)^2^·(OH^−^)^4^	−23.47	[49]

## Data Availability

The data presented in this study are available on request from the corresponding author.
